# Prevalence and associated factors of bronchial asthma among adult patients in Debre Berhan Referral Hospital, Ethiopia 2018: a cross-sectional study

**DOI:** 10.1186/s13104-019-4670-9

**Published:** 2019-09-23

**Authors:** Sisay Shine, Sindew Muhamud, Alebachew Demelash

**Affiliations:** 1 Public Health Department, College of Health Science, Debre Berhan University, P.O.Box: 445, Debre Berhan, Ethiopia; 2 Nursing Department, College of Health Science, Debre Berhan University, Debre Berhan, Ethiopia

**Keywords:** Adult patient, Bronchial asthma, Referral Hospital, Debre Berhan, Ethiopia

## Abstract

**Objective:**

Bronchial asthma is one of the major public health challenges throughout the world that negatively impact patients, families and community. The objective of this study was to assess the prevalence and associated risk factors of bronchial asthma among patients in adult emergency department of Debre Berhan Referral Hospital. A hospital based cross-sectional study design was used among 257 study participants. A systematic sampling technique was used to select the study participants. Data was collected by using pretested and structured questionnaire and analyzed by using SPSS version 20.0. Both bivariate and multivariate logistic regression model was used to identify the predictors of asthma.

**Results:**

Prevalence of bronchial asthma among adult patients was 29.6%. Being an urban resident (AOR: 1.5: 95% CI 1.3–3.9), income of household less than 1000 EBr/month (AOR: 1.7: 95% CI 1.6–4.1), having family history of asthma (AOR: 2.7: 95% CI 1.3–5.8), and presence of vermin in the house (AOR: 2.4: 95% CI 1.2–4.7) were significantly associated with bronchial asthma. The authors concluded that the prevalence of bronchial asthma among adult patients was high. Therefore, educational program about the risk factors and preventive measures of asthma is highly recommended.

## Introduction

Bronchial asthma is one of the most prevalent chronic inflammatory disorders of the airway, which subsequently results an increased contractibility of the surrounding smooth muscles and worsened pulmonary function [1]. It is the major public health challenge throughout the world that negatively affect patients, their families and the community by inducing work and school loss, poor quality of life, frequent emergency visits, hospitalizations and deaths that occur at any age. It also represents a major economic and social burden both in developed and developing countries [2–4].

According to the Center of Disease Control and Prevention report, an estimated 235 million people which include 6 million children have bronchial asthma worldwide [5]. The prevalence of asthma in developing countries increased in 50% per decade for the last 40 years and approximately 250,000 deaths occur in each year. It was the common conditions that affect 5–10% of the population during the past 20 years [6, 7].

Bronchial asthma is one of the most common public health problems in Ethiopia. Its prevalence increased over the last few decades with different contributing factors such cigarette smoking [8, 9], household economic status [10], occupational condition of the patients [9], residence of the patients [11], presence of vermin at household [9] and family history of asthma [12].

Bronchial asthma is being common from year to year; however, it is not having the level of attention it deserves for its proper management. Although its risk factors could be avoided to prevent the occurrence or even the exacerbation in Ethiopia, many health professionals and even the health sectors have done an inefficient effort. The level of awareness of the community toward the prevention of the disorder is suggested to be inadequate. Therefore, this study aims to contribute to filling information gaps on the existing bronchial asthma magnitude and associated risk factors. The data obtained from this study will have its own contribution for policymakers and clinicians to plan and evaluate the management of bronchial asthma.

## Main text

### Methods

#### Study design, setting and population

A hospital based cross-sectional study design was carried out from March 01 to April 30, 2018, among adult patients in an adult emergency department of Debre Berhan Referral Hospital. The hospital is located in Debre Berhan town, 130 km north of Addis Ababa. It is the only governmental referral hospital in the town. It provides both curative and preventive services for 3 million people in its catchment area. All adult patients who visited adult emergency department were our source population.

#### Sample size and sampling strategies

A total of 257 adult patients had participated in the study. Each study participant was selected through systematic sampling technique with an interval of every fourth adult patients who visited the adult emergency department.

#### Operational definition

Bronchial asthma: a study subject was labeled as bronchial asthmatic if he/she has the symptoms of shortness of breath with wheezing and having normal breathing in between episodes of shortness of breath that lasts for 3 months in the past 12 months. Those asthmatic patients whose age < 13 years and critically ill who can’t able to respond were excluded from the study.

Chronic disease: course of the disease that lasts for more than 3 months.

Drug discontinuation: a study participant withdraws drugs in an attempt to improve outcomes for any disease.

Exercise habit: a study participant who has an experience of physical exercise once per a day for more than 30 min for relaxation or health.

Frequent utilization of perfume: a study participant utilizes perfume at their home more than twice per a day.

#### Data collection tool and methods

The structured questionnaire adapted from reviewing different literature of similar studies was used for the collection of quantitative data [2, 12]. Data on bronchial asthma, demographic characteristics, environmental and housing conditions, individual behavior and health-related factors were collected via interview. Data was collected immediately after stabilizing from emergency department. A team of three experienced data collectors were trained in conducting an interview. Interviewer were available to collect data at the daytime only. English version questionnaire was translated into Amharic language and again translated back to English by experts who were fluent in both languages to check consistency. This study was carried out after getting ethical clearance from Debre Berhan University research ethics review committee. Verbal informed consent was taken from the study participants after briefed about the study. Omitting name of the study participants from the questionnaire help to assure confidentiality of the information.

#### Statistical analysis

The data was entered by using Epi-Info 7.0.9.7 version computer software package for editing, cleaning, coding, and checking completeness and consistency and exported to SPSS window version 20.0 for analysis. Descriptive analysis was done to describe the characteristics of our study population. Both bivariate and multivariate logistic regression analysis were used to identify the predictors of asthma. Variables with 95% confidence interval and P value < 0.05 during the bivariate analysis were included in the multivariate logistic regression analysis to see the relative effect of confounding variables. Adjusted odds ratios with 95% confidence interval were calculated and P-value less than 0.05 were considered as statistically significant. Finally, data was displayed by tables, graphs and statements.

### Result

#### Socio-demographic characteristics of the study participants

Two hundred fifty-seven (n = 257) adult patients were enrolled in the study. They made a response rate of 100%. One hundred twenty-four (48.2%) males and 133 (51.8%) females were participated in the study. The mean age of the participants was 38.58 (SD ± 12.81) years old. About 44.7% were living in urban and 66.9% were married by their marital status.

#### Environmental and housing characteristics of the study participants

From the total participants, 38.5% their kitchen attached to the main house. The larger proportion of participants, 91.8% their house have window. About 50.6% households used charcoal as source of energy, followed by electricity 46.7%. Of the total participants, 48.7% have their own house followed by rented from private owner 31.1%. About 43.6% of the participants live with pets.

#### Individual behavior and health-related condition

About 10.5% of the study participants were cigarette smokers. Among the participants, 37.7% had family history of asthma. Fifty-six (21.8%) of the study participants had an experience of pneumonia in the last 12 months. Among the participants, 12.5% had habit of frequent utilization of perfume and 86.4% had no habit of exercise (Table 1).

**Table 1:** Individual behavior and health related characteristics of adult patients in Debre Berhan Referral Hospital, Ethiopia 2018

Variable	Frequency	Percent
Cigarette smoking		
Yes	27	10.5
No	230	89.5
The family history of asthma		
Yes	97	37.7
No	160	62.3
Presence pneumonia in the last 12 months		
Yes	56	21.8
No	201	78.2
Experience of drug discontinuation		
Yes	29	11.3
No	228	88.7
Habit of frequent usage of perfume		
Yes	32	12.5
No	225	87.5
Habit of bronchodilators drug utilization		
Yes	127	49.4
No	130	50.6
Exercise habit		
Yes	35	13.6
No	222	86.4

#### The magnitude of bronchial asthma among adult patients

**Fig. 1:**
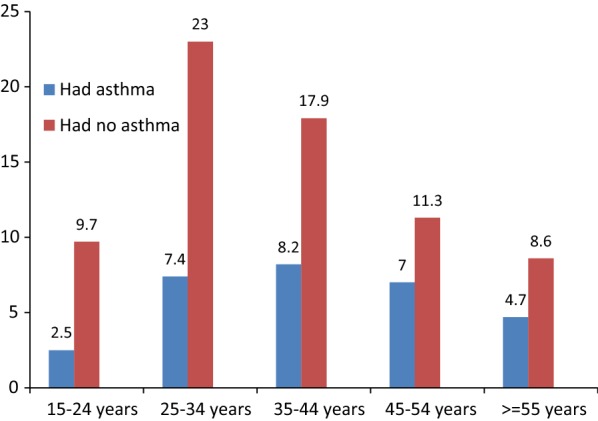
Prevalence of bronchial asthma with age categories among adult in Debre Berhan Referral Hospital, Ethiopia 2018

The prevalence of bronchial asthma among the study participants was 29.6%. Among participants who have bronchial asthma 17.9% were male and 8.2% at the age range of 35–44 years old (Fig. 1). Majority, 16.3% of participants who have bronchial asthma were from urban residence.

#### Factors associated with bronchial asthma

In the bivariate logistic regression analysis, sex of patients, the residence of the patients, monthly income of the households, the house ownership, the presence of vermin in the house, family history of asthma, and habits of bronchodilators drug utilization were found to be significantly associated with bronchial asthma. The result from multivariate logistic regression analysis revealed that being urban resident (AOR: 1.5: 95% CI 1.3–3.9), income of household less than 1000 EBr/month (AOR: 1.7: 95% CI 1.6–4.1), having family history of asthma (AOR: 2.7: 95% CI 1.3–5.8), and presence of vermin in the house (AOR: 2.4: 95% CI 1.2–4.7) were significant predictors of bronchial asthma. But sex, house ownership and habits of bronchodilators drug utilization among patients didn’t show significant association with bronchial asthma (Table 2).

**Table 2:** Factors associated with bronchial asthma among adult patients in Debre Berhan Referral Hospital, Ethiopia 2018

Variable	Bronchial asthma		COR (95% CI)	AOR (95% CI)
	Yes (%)	No (%)		
Sex of adult patients				
Male	46 (17.9)	78 (30.3)	2.0 (1.2–3.5)	0.6 (0.3–1.1)
Female	30 (11.7)	103 (40.1)	1.0	1.0
Residence				
Urban	42 (16.3)	73 (28.4)	1.8 (1.2–3.1)	1.5 (1.3–3.9)*
Rural	34 (13.3)	108 (42.0)	1.0	1.0
Monthly income (in EBr)				
< 1000	17 (6.6)	20 (7.8)	2.9 (1.3–4.1)	1.7 (1.6–4.1)*
1000–5000	36 (14.1)	81 (31.5)	1.6 (0.9–4.1)	1.7 (0.7–3.9)
> 5000	23 (9.9)	80 (31.1)	1.0	1.0
House ownership				
Private	31 (12.1)	94 (36.6)	1.0	1.0
Rented from gov’t	25 (9.7)	27 (10.6)	2.6 (0.2–0.7)	0.4 (0.2–1.8)
Rented from private owner	20 (7.8)	60 (23.3)	1.2 (1.1–1.8)	0.9 (0.5–1.8)
Present of vermin at household				
Yes	58 (22.6)	102 (39.7)	2.5 (1.4–4.6)	2.4 (1.2–4.7)*
No	18 (7.0)	79 (30.7)	1.0	1.0
Family history of asthma				
Yes	38 (14.8)	59 (23.0)	2.1 (1.2–3.6)	2.7 (1.3–5.8)*
No	38 (18.8)	122 (47.4)	1.00	
The habit of bronchodilators drug utilization				
Yes	46 (17.9)	81 (31.5)	1.9 (0.7–2.5)	2.1 (0.9–4.5)
No	30 (11.7)	100 (38.9)	1.0	

* Significant at *P* < 0.05

### Discussion

This institution-based cross-sectional study attempted to assess the magnitude and associated risk factors of asthma among adult patients in an adult emergency department of Debre Berhan Referral Hospital.

In the present study, the prevalence of bronchial asthma among adult patients in the referral hospital was 29.6%. This prevalence was higher compared to the study done in Uganda, Nigeria, Egypt and Ethiopia [13, 14]. A possible explanation for this difference could be due to the effect of climatic conditions. This study had been conducted on participants living in highland area which is elevated 2810 m (9219 fts) above sea level and having very cold climate in almost all months of the year. Furthermore, growing population size and radical urbanization contributes to the increment of the prevalence of asthma by changing the pattern of environmental condition and lifestyle of the community. However, variables that hadn’t been included in the above studies, such as the level of air pollution, level of exposure to allergens, and climatic conditions, may have contributions for the difference of prevalence in different areas. This can be the concern of future researches.

This study revealed that urban residents (AOR: 1.5; 95% CI 1.3–3.90) were more likely to develop bronchial asthma than rural residents. It was consistent with the report in Brazil and Ethiopia [15, 16]. This might be explained outdoor air of urban area is highly polluted due to high levels of traffic and industry related emissions that could increase the risk of asthma. In contrast, study done in India [17] showed that being a rural resident was significantly higher the odds of having asthma. Research conducted in Ethiopia [18] revealed that no association between asthma and residence of the patients. These variations might be due to the difference in the characteristics of the study population, geographical distribution and case definitions used to ascertain asthma.

As of this study, adult patient who come from low income status were more likely to develop bronchial asthma than counterpart. It was congruent with the study conducted in Australia [19]. This could be due to the fact that, low income levels of the households limited to apply appropriate prevention and control mechanisms of bronchial asthma.

This study indicated that patients who come from the family history of asthma (AOR: 2.7: 95% CI 1.3–5.8) were more likely to develop bronchial asthma than from non-asthmatic family. Similar findings have been reported by other studies in developed and developing countries that showed a significant association between family history of asthma and asthma occurrence among adult patients [20, 21]. This association could be either due to genetic factors or a shared environment by the family members.

In this study, present of vermin at household level increased the probability of developing asthma among adult patients. The world health organization report in 2008 report also showed that evidence for a relationship between asthma and domestic exposure to cockroaches, mice and dust mites is strong [22]. This could be explained by house which have vermin’s concerns with the use of insecticides prays at home, with more frequent use being associated with bronchial asthma. Similar results can be found in the literature regarding the link between the use of home aerosolized cleaning products and asthma [23, 24].

### Conclusion

Prevalence of bronchial asthma among adult patient who visited adult emergency department in the study area was high. Being an urban resident, presence of vermin at house and adults from the family of having a history of asthma were the risk to develop bronchial asthma. So, health education program for educating people especially for urban residents and people live in a house which have vermin’s about the risk factors and preventive measures were highly recommended. Further, longitudinal studies are required to investigate possible risk factors of bronchial asthma.

## Limitation of the study

In this study, we have limitations that should be noted. Use of cross-sectional study may not create a true causal relationship between bronchial asthma and its risk factors. Using self-reported data and collecting data at the daytime only might increase or decrease the prevalence of asthma.

## Data Availability

All data supporting the findings are contained in the manuscript. Anyway, datasets are available from the corresponding author on reasonable request.

## Abbreviations

AOR: adjusted odd ratio
EBr: Ethiopian Birr
Gov’t: government
OPD: outpatient department
SD: standard deviation

## References

[1] Masoli M, Fabian D, Holt S, Beasley R (2004). The global burden of asthma: the executive summary of the GINA dissemination committee report allergy. Eur J Allergy Clin Immunol.

[2] Aggarwal AN, Chaudhury K, Chabra SK (2006). Prevalence and risk factors for bronchial asthma in indian adults. Indian J Chest Disord Allied Sci.

[3] Batman ED, Hurd SS, Barnes PJ, Bousquet J, Drazen JM, Fitz GM (2008). Global strategy for asthma management and prevention: GINA excutive summery. Eur Respir J.

[4] Buist AS, Vollmer WM (1990). Reflections on the rise in asthma morbidity and mortality. J Am Med Assoc.

[5] Pearce N, Strachan D (2011). The global asthma report 2011.

[6] Gupta RS, Weiss KB (2009). The national asthma education and prevention program asthma guideline accelerating their implementation and facilitating their impact on children on asthma. Pediatrics.

[7] Braman SS (2006). The global burden of asthma. Chest.

[8] Tefereedgn EY, Ayana AM (2018). Prevalence of asthma and its association with daily habits in Jimma Town, Ethiopia. Open J Asthma.

[9] Wjst M, Boakye D (2007). Asthma in Africa. PLoS Med.

[10] Korinan F, Fanta DF (2016). Asthmatic patients on follow-up at chest clinic of Jimma University. Indo Am J Pharm Res.

[11] Sharma S, Sood M, Sood A (2011). Environmental risk factors in relation to childhood asthma in rural area. Curr Pediatr Res.

[12] Elfaki NK, Shiby AY (2017). Risk factors associated with asthma among Saudi adults in Najran. J Clin Respir Dis Care.

[13] Tageldin MA, Wagih K, Maher O (2015). Study the pattern of bronchial asthma among outpatients clinic at Sohag and Akhmeem Chest Hospitals. Egypt Soc Chest Dis Tuberc.

[14] Kirenga JB, Okot-Nwang M (2012). The proportion of asthma and patterns of asthma medications prescriptions among adult patients in the chest, accident and emergency units of a tertiary health care facility in Uganda. Afr Health Sci.

[15] Sewunet A, Daniel A, Dawit K, Begashaw M (2018). Assessment of predictors for acute asthma attack in asthmatic patients visiting an Ethiopian hospital: are the potential factors still a threat?. Asthma Res Pract.

[16] Felipe MS, Karynna PV, Luciana TS, Evelyn L, Mariana RG, Rafael S, Claudia S (2018). Trend of self-reported asthma prevalence in Brazil from 2003 to 2013 in adults and factors associated with prevalence. J Bras Pneumol..

[17] Gupta PR, Mangal DK (2006). Prevalence and risk factors for bronchial asthma in adults in Jaipur district of Rajasthan (India). Lung India.

[18] Korinan F, Fekede BD (2016). Uncontrolled asthma and associated factors among adult asthmatic patients on follow-up at chest clinic of Jimma university specialized hospital, south-west Ethiopia. Indo Am J Pharm Res.

[19] Hedlund U, Eriksson K, Ronmark E (2006). Socio-economic status is related to incidence of asthma and respiratory symptoms in adults. Eur Respir J.

[20] Dillip KD, Mrutunjaya D, Bibhudatta D, Mamata DM, Swarup KB (2017). Association of dietary pattern on asthma and allergic disorders: an observational hospital based study. Int J Contemp Pediatr.

[21] Shirina A, Abdishakur A, Roos B (2010). Prevalence and risk factors of asthma among adolescents and their parents in Al-Ain (United Arab Emirates). Respiration.

[22] WHO. Public Health Significance of Urban Pests. WHO Regional Office for Europe; 2008.

[23] Le Moual N, Varraso R, Siroux V, Dumas O, Nadif R, Pin I, Zock JP, Kauffmann F (2012). Domestic use of cleaning sprays and asthma activity in females. Eur Respir J.

[24] Annabelle B, Raphaëlle V, Margaux S, Françoise CC, Jan-Paul Z, Francine K, NicoleLe M (2014). Cleaning sprays, household help and asthma among elderly women. Respir Med.

